# Biomolecular and cellular effects in skin wound healing: the association between ascorbic acid and hypoxia-induced factor

**DOI:** 10.1186/s13036-023-00380-6

**Published:** 2023-10-02

**Authors:** Maryam Ghahremani-Nasab, Azizeh Rahmani Del Bakhshayesh, Naeimeh Akbari-Gharalari, Ahmad Mehdipour

**Affiliations:** 1 Department of Tissue Engineering, Faculty of Advanced Medical Sciences, Tabriz University of Medical Sciences, Tabriz, Iran; 2 Department of Neurosciences and Cognition, Faculty of Advanced Medical Sciences, Tabriz University of Medical Sciences, Tabriz, Iran

**Keywords:** Skin wound healing, Ascorbic acid, Hypoxia-induced factor, Fibroblasts, Keratinocytes

## Abstract

The skin serves as a barrier to protect the body from environmental microorganisms and is the largest tissue of the body and any damage must be quickly and effectively repaired. The fundamental purpose of dermal fibroblasts is to produce and secrete extracellular matrix, which is crucial for healing wounds. The production of collagen by dermal fibroblasts requires the cofactor ascorbic acid, a free radical scavenger. In skin wounds, the presence of Ascorbic acid (AA) decreases the expression of pro-inflammatory factors and increases the expression of wound-healing factors. In addition, AA plays an important role in all three phases of wound healing, including inflammation, proliferation, and regeneration. On the other hand, growing evidence indicates that hypoxia improves the wound healing performance of mesenchymal stem cell-conditioned medium compared to the normoxic-conditioned medium. In a hypoxic-conditioned medium, the proliferation and migration of endothelial cells, fibroblasts, and keratinocytes (important cells in accelerating skin wound healing) increase. In this review, the role of AA, hypoxia, and their interactions on wound healing will be discussed and summarized by the in vitro and in vivo studies conducted to date.

**Graphical Abstract:**

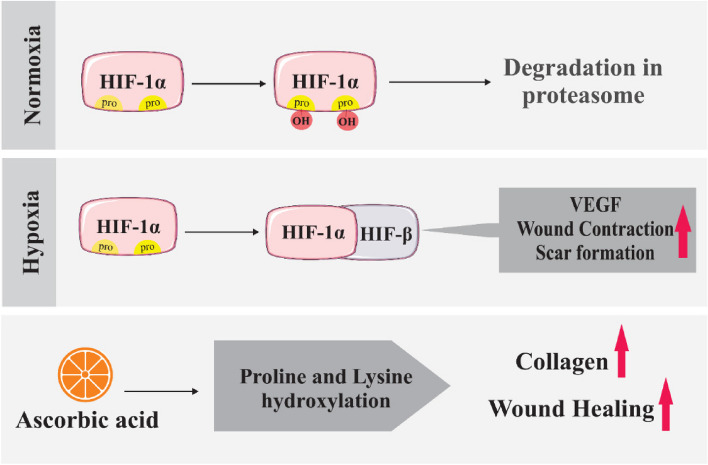

## Background

**Fig. 1:**
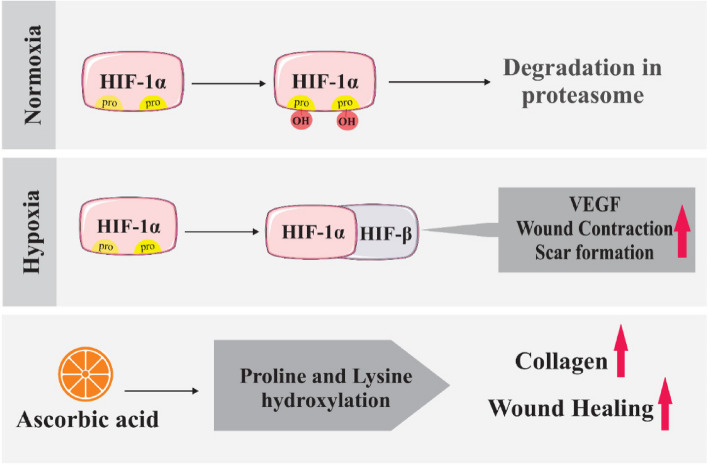
Ascorbic acid (AA) and hypoxia in skin wound healing

The most extensive tissue of the human body is the skin, which consists of two layers, the epidermis and the dermis, and supports the body from the surrounding microenvironment, protects the body from ultraviolet rays, regulates the balance of fluids and body temperature [1–3]. Wound healing is a multifaceted biological process that consists of four consecutive and overlapping programmed phases: hemostasis, inflammation, proliferation, and tissue remodeling. After the hemostasis phase that occurs in the first seconds after wounding and starts with vascular constriction and the fibrin clot formation, inflammatory cells are called to the wound site. The first cells to be recruited are neutrophils, which clean the wound from infectious materials and signal the recruitment of macrophages to clean up bacteria, spent neutrophils, and damaged materials. Moreover, macrophages are thought to coordinate the healing process by signaling fibroblasts to form granulation and remodel tissue at the site of the wound as well as by supplying critical signals for re-epithelialization to repair damaged tissues [4–6]. In the process of re-epithelialization, skin wounds resurface with new epithelium, and the skin’s barrier function is restored [2, 7]. It is thought that re-epithelialization of full-thickness and partial-thickness wounds varies based on the origin of the cells involved in the healing process. In partial-thickness wounds, basal stem cells of the interfollicular epidermis participate to a lesser extent, instead, stem cells of eccrine sweat glands and pilosebaceous units are more involved. However, in the case of full-thickness wounds, these sections have been damaged, and interfollicular epidermal cells located at wound borders participate. During the proliferation phase, about 16–24 h after injury, re-epithelialization starts, and cutaneous cells and epidermal keratinocytes located close to the wound site, produced cytokines, chemokines, and growth factors leading to a change in keratinocytes phenotype so keratinocytes become activated and proliferate and migrate into the wound site [2, 4]. Also, the proliferation and migration of fibroblasts from multiple sources occur to the restoration of the underlying dermis layer, clearing of the wound site from fibrin clot, and replacing it with collagen matrix. Furthermore, rearrangement of collagen fibers and wound contraction occurs in the presence of fibroblasts. Fibroblasts, macrophages, and keratinocytes produce growth factors that initiate the process of blood vessel proliferation [4]. Repair and regeneration are two healing ways for cutaneous wounds and these two healing mechanisms are different from each other. In the general explanation, healing tissue in wound repair, in comparison with intact tissue has inferior characteristics, whiles healing tissue in wound regeneration has the function and morphology of the original one and is an exact copy of intact tissue [8]. Scar formation is the final outcome of the healing process. The scar is fibrous tissue made primarily of unidirectional layers of collagen rather than the basket-weave pattern that is typically observed in normal epidermis. Additionally, the skin strength at the site of repair is typically weaker than that of the intact skin. Hypertrophic and keloid scars are two similar variations of the scar. It has not been possible to prevent scar formation at this stage entirely with any intervention; instead, the size of the scar can be minimized. Recently, many types of research in the field of natural and impaired wound healing and vital factors affecting them have been done, which may lead to the emergence of effective treatments in the process of proper wound healing and promotion [4, 9–15]. There is a belief about the importance of nutritional support to regenerate injured skin layers [4, 13, 14, 16, 17]. One of these nutrients is ascorbic acid (AA), which is present in substantial amounts in normal tissues. It has been considered in the discussion of wound healing because its amount is insufficient in the damaged tissue [18, 19]. According to the studies, it can be underlined that AA is responsible for numerous fundamental functions, including participation in the construction of tissues, raising immunity and maintaining skin vitality [20]. Some physiological responses of the body also affect the wound-healing process. Among these responses, local hypoxia, which is a physiological response to the wound, plays an important role in defining the success of the natural healing process. It can be said that HIF-1 (Hypoxia-inducible factor-1) is the main regulator of oxygen homeostasis and acts as the main determining factor of healing outcomes and it contributes to all wound healing phases through its role in cell survival under hypoxic conditions, cell migration, and proliferation, synthesis of the matrix, and release of growth factors throughout the healing process (Fig. 1) [21]. Therefore, a general overview of the possible effects of AA and hypoxia on wound healing is necessary. Thus, this article reviews the relationship between AA and hypoxia in skin wound healing and addresses the relevant molecular mechanisms.

## Ascorbic acid

### Chemical structure, functions, absorption, and transporters

**Fig. 2:**
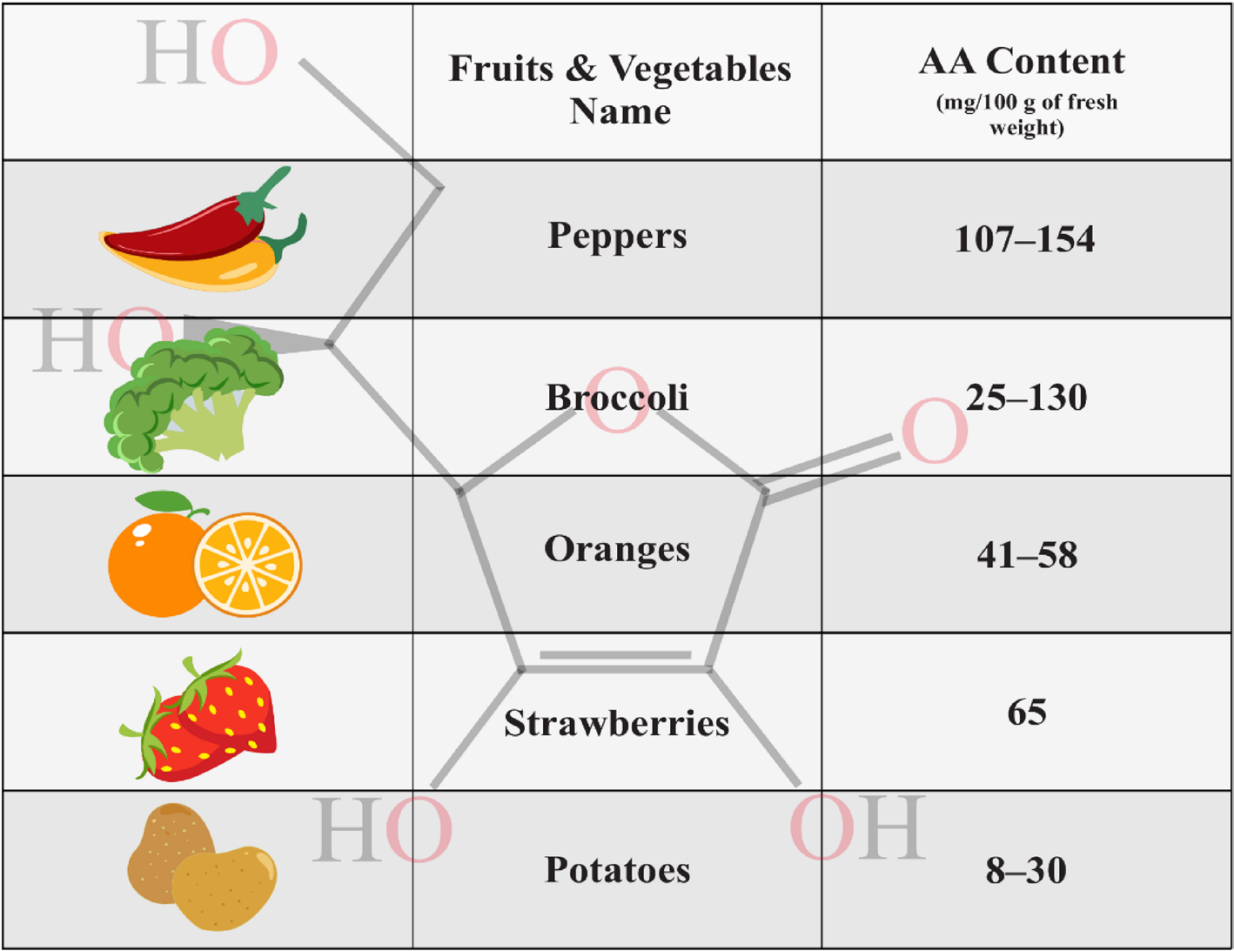
The chemical structure of L-ascorbic acid and its most important sources [25]

Any severe wound can lead to a catabolic state. After wounding, there is a significant increase in the rate of micronutrient metabolism, resulting in critical deficiencies. Also, level of AA drops quickly during inflammation. Ascorbic acid (AA, vitamin C) (Fig. 2), as a water-soluble micronutrient, is one of these nutrients and is essential for the function of healthy tissues and organs as well as in the process of tissue repair and regeneration. The L-gulono-lactone oxidase (GLO) gene, for the final step of the biosynthesis of ascorbate from glucose, was inactivated in human ancestors 61 million years ago, leaving these species dependent on exogenous AA (from the diet). Conversely, the majority of animals can synthesize AA from glucose in their kidney or liver. As well as this gene is conserved in wild‐type mice and has the capacity to biosynthesis of AA within their tissues [4, 22–26]. Furthermore, the stability of AA is influenced by pH, with optimal stability observed in the pH range of 4–6 [27]. Within the human body, L-dehydroascorbic acid is an oxide form of AA, can readily convert to L-ascorbic acid (biological aspects of AA), and under physiological pH conditions, it is almost completely in the form of an ascorbate anion due to its low pKa value (pKa = 4.2). AA is synthesized through a two-step reaction, generally from d-galacturonic acid or l-galactose as starting materials. The physicochemical properties of AA are determined by its chemical structure, which includes two distinct forms: an epimer and an enantiomer, known as D-araboascorbic acid (or ascorbic acid, recognized as a food additive) and D-ascorbic acid, respectively. The preservation of AA within human tissues is limited, and the body rapidly eliminates it [18, 20, 25, 26].

**Fig. 3:**
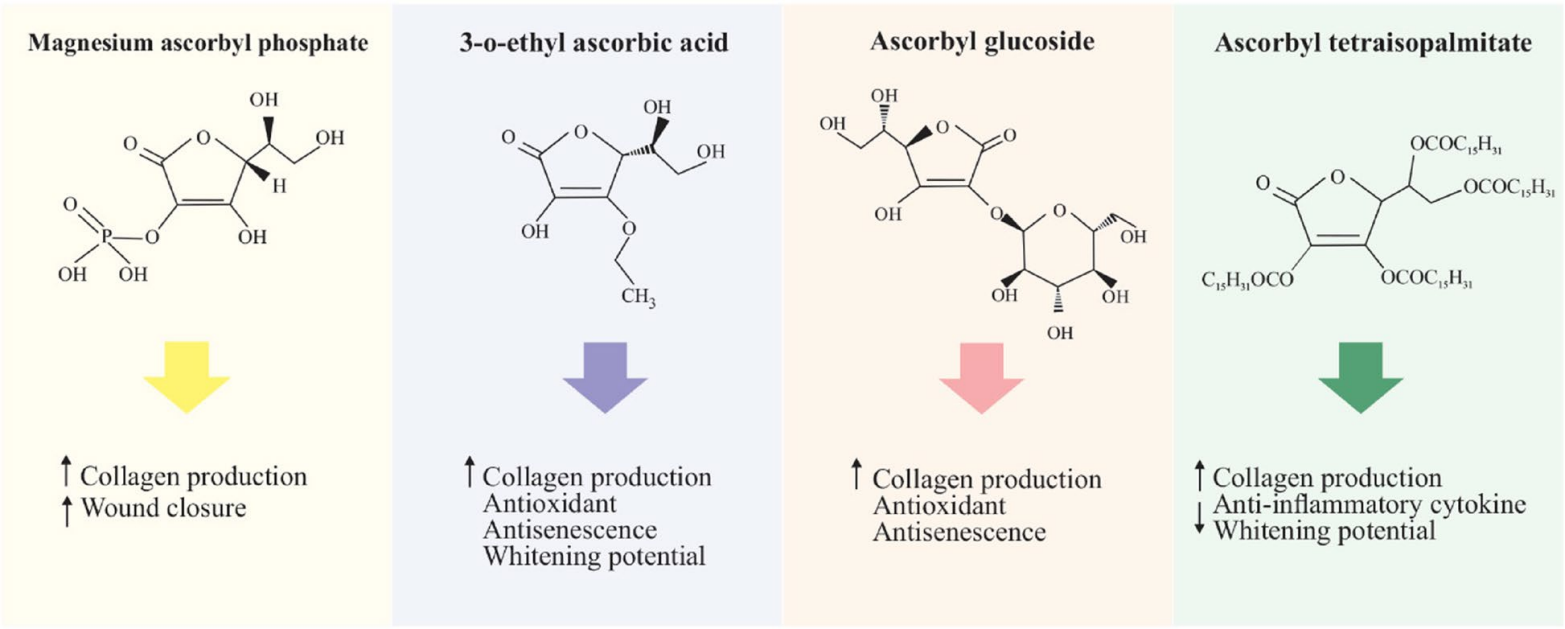
AA derivatives that promote wound healing

Many physiological functions require various organic substances such as ascorbate as antioxidants and cofactors in certain enzymatic pathways involved in numerous cellular activities [19, 26, 28]. AA acts as an electron donor and decreases reactive oxygen species (ROS) such as hydroxyl radicals and superoxide radicals [19]. The enzymatic hydroxylation of lysine and proline, which is catalyzed by dioxygenase enzymes, is dependent on the presence of AA. As well AA promotes the secretion of procollagen containing hydroxyproline, also it serves as a crucial co-factor in the biosynthesis of collagen, metabolism of catecholamine and carnitine, as well as in the absorption of dietary iron [19, 29]. Furthermore, AA is a crucial nutrient for the proper function of skin collagen synthesis and differentiation of keratinocytes [4], in addition, after in vitro ultraviolet B (UVB) irradiation, it has been shown that AA can protect keratinocytes from damage by ROS [4]. AA through the reduction of dopaquinone to dopa, a key substrate in the melanin biosynthesis pathway, causes inhibition of melanin production in melanocytes, so AA has an important role as a depigmenting agent [19]. Figure 3 depicts the effects of various AA derivatives, including Magnesium ascorbyl phosphate [30–32], 3-o-ethyl ascorbic acid [30–34], Ascorbyl glucoside [30, 31], and Ascorbyl tetraisopalmitate [30, 31, 35], on wound healing and tissue repair. There are two mechanisms by which AA can be absorbed into the body: passive diffusion within the oral cavity or active sodium-dependent vitamin C transporters (SVCT) within the gastrointestinal tract [20, 36]. SVCT1 and 2 are two specialized and important transporter isoforms responsible for the transportation of AA through cell membranes in the intestine and especially for its reabsorption in the kidney [26]. In fibroblasts that synthesize collagen, SVCT2 is an important transporter in the uptake of AA from the extracellular fluid [19]. Most of the tissues express SVCT2 only, in contrast to the epidermis which expresses both SVCT1 and SVCT2 transporters [4], nevertheless, renal and gastrointestinal absorption of AA happens just from SVCT1 transporter [37]. Due to the high physiological importance of these transporters, mice with the SVCT2 knockout, die instantly upon birth [26]. SVCT1 transporters are located on the apical surface of epithelial cells and facilitate the uptake of both dehydroascorbic acid (DHA) and AA from the gastrointestinal tract. Moreover, SVCT2 transporters are situated on the basolateral surface of these cells. This suggests that ascorbate is extracted from epithelial cells via both interstitial fluids and the gastrointestinal tract. As a result, there is no effective enforcement mechanism to ensure that ascorbate fluxes into the blood circulation through the gastrointestinal tract [36]. The AA concentration in normal skin is notably higher compared to other tissues and plasma, so the dermal layer cells receive the necessary amount of AA through blood circulation. Apparently, in the skin, the intracellular compartments exhibit the highest amount of AA, and this amount has been measured at its highest concentration, in the range of millimolar (concentration inside the cell ~ 1–10 mM and in the blood plasma ~ 50 μM) [18, 24]. It has been reported that oxidant stress caused by UV irradiation and pollutants is associated with depleted levels of AA in the epidermis. Without a doubt, the concentration of epidermis AA is greater than that of the dermis (differences of 2-5-fold) [4]. When taking AA orally, an insufficient amount of it delivers to the skin; as a result, it will not have proper bioavailability, and the oral route is not recommended as a suitable source to supply the necessary amount of AA to peripheral structures such as the skin. The important method for providing AA to the skin is through local or topical application. Studies have also shown that the topical application of AA improves surgical wound healing and promotes tissue reconstruction [18, 38].

However, there have been reports regarding contact dermatitis resulting from the consumption of cosmetics products containing AA derivatives (such as ascorbyl tetraisopalmitate and 3-O-ethyl ascorbate, used in some cosmetic products) for topical application. In a study done by Belhadjali et al., it was demonstrated that oral AA formulation is more tolerable than topical formulation, which is used in anti-aging cosmetic products, and the use of AA in such cosmetics causes allergic contact dermatitis. It is also reported that topical formulations of ascorbyl tetraisopalmitate in an anti-aging lotion are used to treat atopic dermatitis [18]. AA is usually considered non-toxic and safe even at high doses over a long period, partly because of its solubility in water. The recommended daily dose of AA in the U.S. (≥ 100 times) is deemed safe, and in well-designed studies, no adverse effects have been observed associated with this daily dose, and any extra consumption of AA is excreted in the urine from the body. Hence, the property of water solubility and excretion in the urine show why AA and water-soluble vitamins rarely cause toxicity. Some studies have shown that AA consumption within the range of 1000–1500 mg per day may cause side effects such as flatulence, diarrhea, and gastric pain. Nevertheless, laboratory investigations have not shown the negative effects and toxicity risks of AA with high levels of consumption. The use of AA also has high positive health benefits compared to the potential risks of toxicity associated with high levels of use [18].

### The pivotal role of ascorbic acid in collagen synthesis

AA has a significant impact on all levels of wound healing; it contributes to neutrophil clearance during the inflammatory phase, as well as, it assists in collagen synthesis and maturation during the proliferative phase [28]. Collagen is one of the most abundant proteins in mammals, and it is fundamental to the organization and formation of a contiguous inter-stitium all over the epidermis. The production of collagen in the skin is accomplished mostly by dermal fibroblasts, which leads to the formation of the dermal collagen matrix and the basement membrane [38]. It constitutes the essential protein of tendons, bones, skin, blood vessel walls, the cornea, and other connective tissues. Moreover, the main factor of skin elasticity is related to the presence of collagen which is a major component of the extracellular matrix of the dermis [39]. Several in vitro studies have demonstrated the vital effect of AA on the collagen hydroxylase enzymes, and studies have revealed that in the absence of AA in the fibroblast cells, the synthesis and crosslinking of these enzymes are reduced. Similarly, hydroxylysine plays an essential role in the cross-link formation of collagen, and the absence of AA results in structural inconstancy, also AA stimulates gene expression of collagen [4]. On the other hand, AA is important to maintain the active form of lysyl and prolyl hydroxylase enzymes, which facilitate the hydroxylation of lysine and proline with AA serving as a co-factor [19, 40]. One of the many studies conducted on the effect of AA on collagen synthesis is the survey conducted by Kishimoto et al. in 2013. They utilized human skin fibroblasts and in vitro AA exposure to examine the long-term impact of AA on collagen expression (for 120 h). As mentioned, they conducted a long-term culture (120 h, human cells) in the presence of AA, and the results demonstrated that the expression of SVCT2, type 1, and type 4 collagen mRNA was enhanced, as well as an increase in type 1 procollagen synthesis. Consequently, the outcomes of these studies suggest that exposure of human skin fibroblasts to AA over time causes an increase in levels of SVCT2 and type 1/type 4 collagen mRNA expression and synthesis of type 1 procollagen [19]. Thus, it can be asserted that AA stimulates the production of collagen mRNA by fibroblasts [4], and its activity is important for the transformation of procollagen to collagen, in addition to being a pivotal epigenetic enzyme [24]. Gref et al. in 2020, due to improved diffusion and delivery of AA through the barrier of epidermis stratum corneum, conjugated AA to squalene (SQ) covalently (developing a lipophilic form of AA) and conducted an ex vivo study on human skin for 10 days. Results indicate that in human skin, AA-SQ significantly increased epidermal thickness and improved the synthesis of collagen III (the main cutaneous collagens) [38]. Maione-Silva et al. in 2019 encapsulated AA in vesicles with different lipid compositions. They discovered that negatively charged liposomes exhibited a higher tendency to retain AA within the skin. Their investigation revealed that AA enclosed inside the liposomes increased the synthesis of type I collagen in fibroblasts and enhanced UVA-induced damage regeneration in keratinocytes [41].

### Epigenetic regulation of ascorbic acid on wound healing

**Fig. 4:**
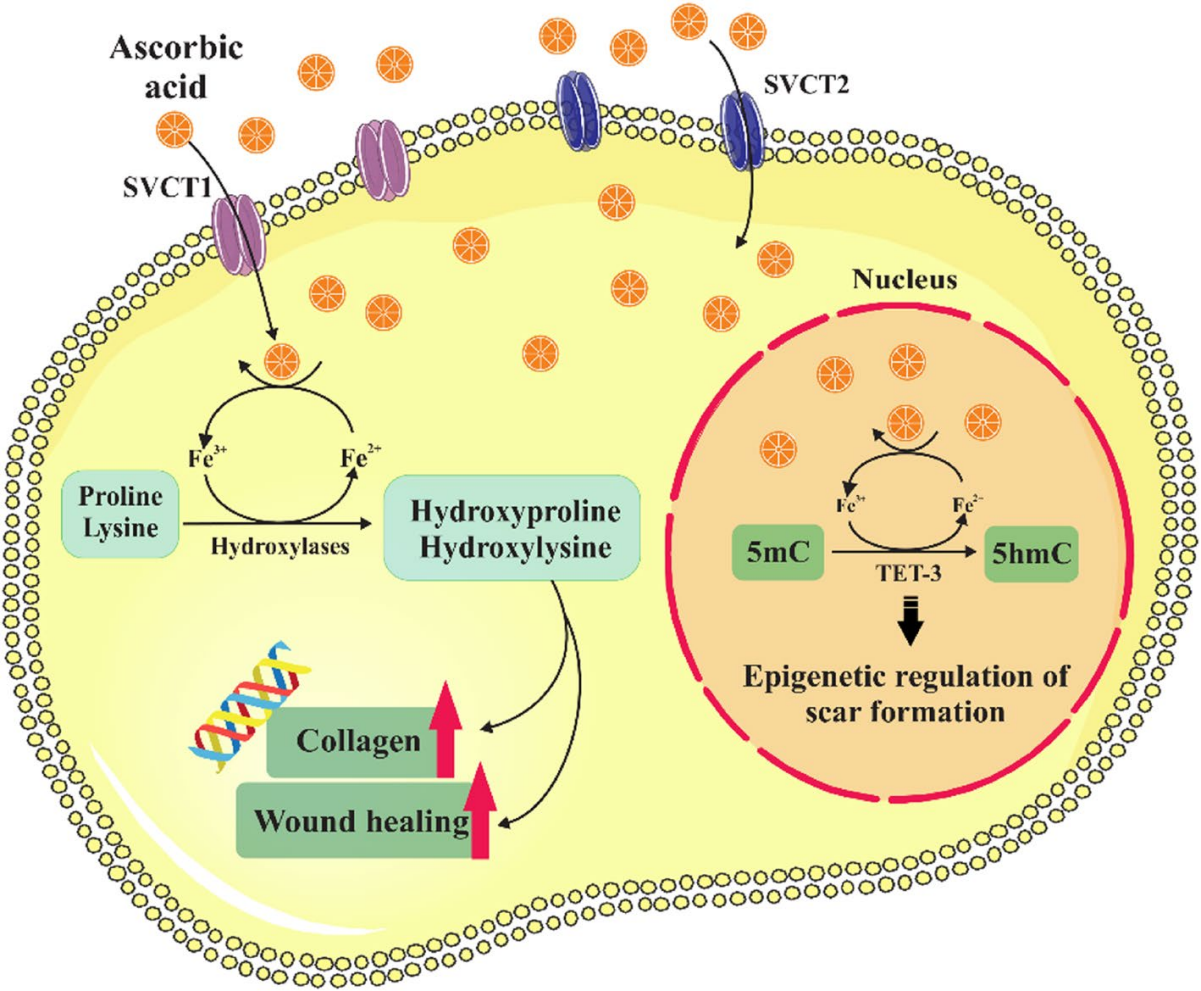
The role of AA on wound healing and epigenetic regulation of scar formation

AA performs substantial biological functions related to maintaining skin health, which can be seen from its abundant concentration in the skin when compared to other human tissues (the epidermis contains between 6 and 64 mg of AA per 100 g of fresh weight, while the dermis contains about 3 and13 mg of AA per 100 g of fresh weight) [18]. Furthermore, in some studies, the participation of AA in the modulation of the immune system has been mentioned [42], and is involved in apoptosis and the clearance of neutrophils during the inflammatory phase of wound healing, along with other levels of wound healing. As mentioned, AA has an important role in collagen synthesis, maturation, secretion, and degradation during the proliferative phase. Several enzymes, such as prolyl hydroxylase and lysyl hydroxylase require AA as a co-factor. Therefore, AA has an important role in the stabilization of collagen and is vital in wound healing [4, 18]. Because of its potent antioxidant properties, AA plays an important role in enzymatic reactions and recent research has demonstrated that it suppresses pro-inflammatory processes and encourages pro-resolution and anti-inflammatory effects in macrophages through pleiotropic mechanisms [23]. AA levels in plasma and tissue drop after wounding; therefore, fibroblasts produce unstable collagen, and collagen maturation is disrupted, leading to impairs wound healing and scarring [18, 42]. Compared to healthy adults, elderly individuals exhibit reduced levels of AA in their plasma, and considering the effect of AA on the proliferation and migration of skin fibroblasts, it can be suggested that administering AA supplements to the elderly individuals may prevent the decrease in the proliferation capacity of fibroblast cells and promotes wound healing in middle-aged adults [4, 18]. The impact of prolonged exposure to ascorbic acid 2-phosphate (AA2P), a stable AA derivative, on contact-inhibited populations of primary human dermal fibroblasts was examined in vitro by Duarte et al. in 2009. The results of the gene expression profiling conducted in this study showed that AA2P and AA modulated cell cycle progression and induced the post-confluent growth of contact-inhibited fibroblasts. The effects of AA2P on the activation of quiescent fibroblasts via cell motility and the presence of serum factors during the process of wound healing are consistent with changes in gene expression. DNA that had been oxidatively damaged displayed quick repair in fibroblasts treated with AA2P. The study reports that AA may increase fibroblast migration and proliferation, thereby enhancing skin protection [43]. Konerding et al. conducted a different study in 2012. The researchers employed diabetic mouse as an incisional wound-healing model to examine the effects of AA, stanozolol, TGF-β (transforming growth factor-β), and copper peptide. In this study, which focused on laparotomy, these agents were transported by a hydrogel, and after a single dose of the hydrogel was applied, the linea alba closure was carried out. Collagen type III contents were significantly higher in the incisional wound groups treated with AA on the 14th day following surgery and demonstrate the beneficial effects of AA applied topically on wound healing [44]. The use of topical silicone gel containing AA to lessen scarring in 80 Asian patients who had facial scars resulting from surgery was studied by Yun et al. (2013). They discovered that applying silicone gel containing AA topically to facial skin improved the aesthetic appearance of surgical scars and minimized their visibility. Evidence suggests that the elevation of scars decreases after the removal of the sutures and continues to decrease in the 2^nd^ and 6^th^ month after surgery, but during this period, erythema is persistent [45]. In a 2018 study, Voss et al. examined the antibacterial and wound healing capabilities of cellulose-based films loaded with AA and/or propolis in a streptozotocin (STZ)-induced diabetic mouse model. The study involved creating a 9 mm incision on the back of each mouse. The diabetic mouse group that received no treatment showed impaired wound healing, whereas the group that received treatment with Cel-PVA/AA and Cel-PVA/AA/Prop films displayed a favorable response in terms of scar formation [22]. As a result, taking everything into account, the studies carried out suggest that using supplements may improve the process of wound healing [42]. Figure 4 illustrates the impact of AA on wound healing via cytoplasmic pathways and its influence on epigenetic regulation. Additionally, Table 1 shows a summary of important studies conducted on the possible influence of AA on the process of wound healing in the past five years.

**Table 1:** An overview of significant studies investigating the potential impact of AA on wound healing

**Agents**	**Wound healing Model**	**Molecular mechanisms**	**Result**	**Year**	**References**
Ascorbic acid, Epigallocatechin gallate, gelatin and chitosan nanoparticles (EV NPS)	In vivo: diabetic mice model	Increased collagen production	Promote wound healing: increasing collagen and angiogenesis, decreasing the inflammatory cells infiltration	2020	[46]
Ascorbic acid, Glycinamide	In Vitro: human dermal fibroblasts	1- AA increased the mRNA level of procollagen COL1A1 and COL3A1 and the protein level of collagen I 2- Glycinamide increased the protein levels of collagen I and III	The combination of AA and glycinamide: increasing collagen production and wound closure	2022	[31]
Ascorbic acid	In vivo: rat pulpotomy model	Increases the expression of SVCT2 and GLUT1 in odontoblasts (acts as AA transporter)	Role in differentiation of odontoblast-like cell and anti-inflammatory response during dental pulp wound healing	2023	[47]
Ascorbic acid	In Vitro: gingival fibroblast	Increases the expression of COL1, FN, IL-6, and bFGF (which are related to fibroblast wound healing activity)	Accelerating the migration of fibroblast cells	2020	[48]
Ascorbic acid 2-glucoside (AA2G)	In Vitro: Bone marrow mesenchymal stem cells (BM-MScs)	Regulating TET2 and increasing the expression of VEGF by activating the PI3K/AKT pathway	Increasing angiogenesis and enhancing the proliferation of BM-MSCs, as well as increasing the speed of wound healing	2022	[49]
Ficoll and Carrageenan in combination with ascorbic acid	In Vitro: iPSCs	Ficoll + AA: Increased collagen deposition Carrageenan: No effect on the amount of collagen, increased deposition of glycosaminoglycan	Boosting collagen and/or GAG deposition and scale up matrix production	2021	[50]
Ascorbic acid, Zinc-based nanoparticles	In Vitro	Antibacterial activity	Speed up wound Healing	2022	[51]
Electro-spun nanofiber containing ascorbic acid and caffeine	In vivo: rat model with skin excision	Collagen deposition	In dressings containing caffeine compared to ascorbic acid: increase of blood vessels and fibroblasts	2019	[52]

## HIF-1 alpha and wound healing

**Fig. 5:**
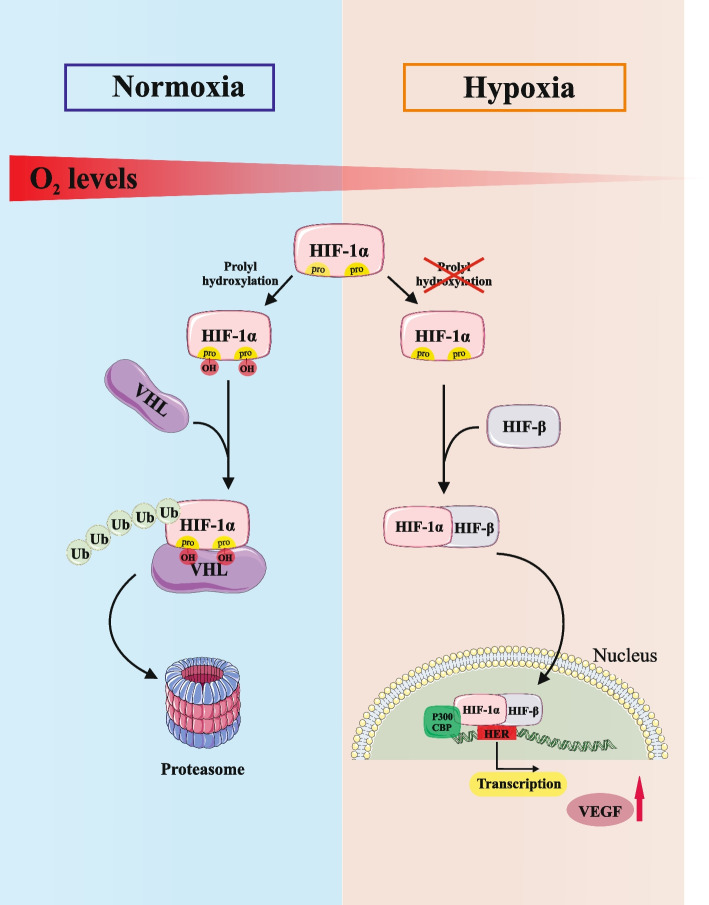
Summary of the HIF-1α pathway in hypoxia and normoxia conditions

Biocompatible and biodegradable scaffolds are used in tissue engineering to support cell culture with the goal of regenerating tissue or organ, leading to selective and limited progress in the repair of some tissues [53, 54]. The potency of tissue engineering to treat complex tissue is severely restricted by difficulties with delivering oxygen and nutrients to developing tissues and removing waste products from developing tissues. The main issue in tissue engineering is that the cells located in the deep regions of the scaffold face a lack of oxygen (anoxia) or reduced oxygen levels (hypoxia), which causes damage to active cells with a high metabolism. In order to ensure that angiogenesis proceeds quickly following the scaffolds are implanted in the patient’s body, it is crucial to provide pre-vascularized scaffolds that already contain blood vessel networks in vitro or to create scaffolds that contain blood vessel-generating cells, matrix composition, and growth factors. Unfortunately, due to the complexity of angiogenesis, researchers have not yet succeeded in achieving this goal [54]. The formation of microvascular networks is a crucial and fundamental aspect of both tissue engineering and wound healing. It can be inferred that hypoxia and the transcription factor hypoxia-inducible factor-1 (HIF-1) strongly influence the regulation of angiogenesis [54]. HIF-1 is crucial for basic cellular metabolism and regulates cellular response to reduced oxygen levels. Additionally, it affects the gene regulation of enzymes and proteins involved in apoptosis, angiogenesis, glycolysis, and the transport of iron. There is also evidence indicating that HIF-1 is present in the hypoxic core of tumors; Therefore, their relationship to cancer has drawn a substantial amount of attention. HIF-1 is organized structurally into an αβ heterodimer whose α subunit is organized by posttranslational modification [55, 56]. HIF-1 is regulated through two different pathways in hypoxic and normoxic conditions. In normoxic conditions, HIF-1 cannot be detected as it binds to the von Hippel-Lindau (VHL) protein at specific proline residues through prolyl hydroxylase, causing in ubiquitination and sending the protein to the proteasome for degradation, and factor-inhibiting HIF(FIH) through asparagine hydroxylation prevents the accumulation of a viable transcriptional complex [55–62]. Under hypoxic conditions, HIF-1α binds to various components such as ARNT, CREB, and EP300 and forms a transcriptional complex that upregulates the transcription of more than 100 genes involved in cell proliferation, apoptosis, glucose metabolism, and angiogenesis (Fig. 5) [57].

In 2014, Chen et al. investigated the impact of normoxic and hypoxic cell-culture conditions on the expression and secretion of paracrine molecules derived from BM-MSCs (such as chemokines, cytokines, and growth factors), which are thought to aid in both in vitro and in vivo cutaneous wound healing. In this study, 18-mm round full-thickness excisional skin wounds on Balb/c nude mice were created and a sealed chamber was used to induce hypoxia. The effects of hypoxia and normoxia on BM-MSCs, as well as their conditioned medium fractions was assessed through ELISA and RT-PCR analyses. The findings revealed that the BM-MSCs expressed and secreted elevated levels of bFGF (basic fibroblast growth factor), VEGF-A (vascular endothelial growth factor A), IL-6 (interleukin 6), and IL-8 (interleukin 8) compared to other cell types. Additionally, the use of hypoCM (hypoxic BM-MSC-derived conditioned medium) compared to norCM (normoxic BM-MSC-derived conditioned medium) significantly improved the proliferation and migration of endothelial cells, fibroblasts, and keratinocytes, as well as enhanced the migration of monocytes, and tubular structure formation by endothelial cells.

Based on the outcomes of in vivo investigations carried out on Balb/c nude mice treated with hypoCM in comparison to norCM, there is a notable acceleration in the skin wound contraction. Collagen I and collagen III levels were significantly lower in the hypoCM-treated group. Conversely, the hypoCM-treated group displayed a marked increase in neovascularization, in vivo cell proliferation, and the recruitment of inflammatory macrophages. These findings suggest that BM-MSCs facilitate skin wound healing through hypoxia-enhanced paracrine [63]. Considering that hypoxia stimulates MSC-CM wound healing properties of MSC-CM (mesenchymal stem cell-conditioned medium), it is interesting to investigate the molecular and cellular mechanisms responsible for the improvement of wound healing function [64]. Therefore, AF-MSCs (human amniotic fluid-derived mesenchymal stem cells), under hypoxic conditions, secrete more paracrine factors related to cell proliferation and survival, according to a study conducted in 2014 by Jun et al. This study examined the proliferation of AF-MSC for three days in both normoxic (20% O2) and hypoxic (1% or 5% O2) conditions. In this study, the proliferation of AF-MSC was investigated under conditions- normoxia (20% O2, 5% CO2) and hypoxia (1% or 5% O2). The findings demonstrated that hypoxia increased the proliferation of AF-MSC cells and preserved their differentiation potential. In vitro studies showed that AF-MSCs cultured under hypoxic conditions secreted higher levels of the paracrine factors TGF-1 and VEGF, as compared to those cultured under normoxic conditions (AF-MSC-norCM) [64]. In addition, in vivo studies have shown that AF-MSC-hypoCM can promote wound closure compared to AF-MSC-norCM in skin injuries [64].

## The effect of ascorbic acid on HIF-1α

AA is a crucial component of both the cytoplasm and nucleus, as was already mentioned. Ascorbate-dependent dioxygenases in the cytoplasm participate in hypoxia response regulation and metabolic processes. AA is required for the oxidative demethylation of 5-methylcytosine in DNA by TET proteins and the removal of the methyl groups from histone lysine by JmjC (Jumonji C) demethylases, both of which occur in the nucleus. AA deficiency has a particularly negative impact on differentiation and subsequent cellular reprogramming processes, such as DNA demethylation [26, 65–72]. As previously mentioned, AA is recognized as a highly functional and significant micronutrient needed for a variety of biological processes, specifically as a crucial enzyme cofactor, and prolyl hydroxylases are one of these enzymes which play an important role in the biosynthesis of collagen and the downregulation of HIF-1 [73–84], and also proline and asparagine hydroxylases control the HIF-1 transcription factor activity through hydroxylation [24, 55]. Inhibitors of the ascorbate-dependent HIF pathway may provide alternative strategies for managing tumor progression, inflammation, and infection [73]. The impact of AA on HIF-1 activation was demonstrated in a 2007 study by Vissers et al. The research involved monitoring the hypoxic response in two primary cell lines in the presence and absence of AA and comparing these results to a cell line that originated from a human tumor. Under normoxic conditions, Intracellular AA concentration is typically low and HIF-1α is present at basal levels. However, when AA was added to the medium at concentrations ranging from 10 to 25 μM, HIF-1α was effectively eliminated [55].

Furthermore, AA plays a role in the epigenetic regulation of gene expression by functioning as a co-factor for the ten-eleven translocation (TET) family of enzymes, which catalyze the hydroxylation of 5mC (5-methylcytosine) to 5hmC (5-hydroxymethylcytosine), and also acts as an intermediary in the process of DNA demethylation (Fig. 4). 5hmC appears to functions as an epigenetic marker and possesses its own transcriptional regulatory activity [4]. It is believed that aberrant epigenetic changes have an important role in the progression of cancer, and some research has revealed that a loss of 5hmC happens through the early progression and development of melanoma [4]. It is interesting to note that AA treatment in melanoma cell lines results in an increase in 5hmC content and causes an alteration in the transcriptome and also a reduction in malignant phenotype. Because AA is vital to maintain the enzyme activity of TETs, this causes the provides additional mechanisms by which AA can influence both cell function and gene expression [4]. In this regard, Lin et al. in 2014 investigated the ability of ascorbic acid (AA) to counteract UV radiation-induced apoptosis. Their findings demonstrated that AA protects epidermal cell lines against UV-induced apoptosis through a TET-dependent mechanism, whereby increasing the expression of p21 and p16 gene [85]. Recent research indicates that hypoxia accelerates the healing of cutaneous wounds by inducing HIF-1α and increasing skin wound contraction [63], thus, it can be concluded that hypoxia may cause scar formation as well as contribute significantly to the excessive fibrosis that characterizes keloid and hypertrophic scars. In the regulation of fibrosing processes, the epigenetic pathway has the main role, based on this, in 2019, Liu et al. evaluated the DNA hydroxymethylation (5-hydroxymethylcytosine; 5-hmC) status in patient scars. Their study showed a significant reduction in scar fibroblasts. In the in vitro study, wherein they cultured human fibroblasts exposed to a known stimulator of HIF-1α, cobalt chloride (CoCl2). Similar to the process in which naturally occurring scars, HIF-1α also leads to the loss of 5-hmC through the downregulation of converting enzymes of 5-hmC known as TETs and also leads to increases in the expression of p-FAK (phosphorylated focal adhesion kinase), which is a crucial mediator of wound contraction. Supplementation of the medium with AA to the medium partially reversed the aforementioned effects, as AA is known as an epigenetic regulator and can minimize excessive scar formation as well as enhance the regenerative healing response [86].

## Conclusion

The crucial process of wound healing has a complicated mechanism involving numerous cells and cytokines. AA is a biological compound that is essential for the metabolism of collagen and for controlling the equilibrium between collagen and elastin in skin fibroblasts. Findings have demonstrated that keratinocyte exposure to AA results in cell differentiation and stratum corneum formation. Furthermore, research conducted both in vivo and in vitro has demonstrated that AA minimizes damage caused by harmful ultraviolet (UV) radiation in keratinocytes. As a result, this biological compound has significantly aided in the healing of skin wounds and reduced the appearance of obvious scars. Collagen is the primary constituent of the extracellular matrix, and as was already mentioned, its production is thought to be a crucial component of the healing process for wounds. However, excessive deposition can result in scarring. The results also show that the induction of hypoxia results in cell proliferation, macrophage attraction to the damaged area, and an increase in angiogenesis, which affects the synthesis and destruction of collagen at the wound site. It also increases wound contraction and accelerates the wound healing process. In addition to accelerating wound healing, hypoxia can cause scarring at the wound site. Scarring, particularly on the face, can negatively impact a person’s psychological well-being and social interactions. Based on the findings, it can be asserted that HIF-1α decreases the expression of TET enzymes, resulting in a reduction in 5-hmC and an up-regulation of p-FAK expression, leading to an enhancement in wound contraction. However, the mentioned effects can be reversed by AA, which acts as an epigenetic regulator. In addition to all the findings, at the moment, there is not sufficient data to prove that AA decreases HIF-1α activity levels through which pathways. One area of future work will be to investigate the 5-hmC/TET3 pathway is warranted to address this gap in knowledge.

## Data Availability

Not applicable.

## Abbreviations

AA: Ascorbic acid
HIF-1: Hypoxia-inducible factor-1
GLO: L-gulono-lactone oxidase
ROS: Reactive oxygen species
UVB: Ultraviolet B
SVCT: Sodium-dependent vitamin C transporters
DHA: Dehydroascorbic acid
AA2P: Ascorbic acid 2-phosphate
TGF-β: Transforming growth factor-β
STZ: Streptozotocin
BM-MScs: Bone marrow mesenchymal stem cells
VHL: Von Hippel-Lindau
FIH: Factor-inhibiting HIF
bFGF: Basic fibroblast growth factor
VEGF-A: Vascular endothelial growth factor A
IL-6: Interleukin 6
IL-8: Interleukin 8
hypoCM: Hypoxic BM-MSC-derived conditioned medium
norCM: Normoxic BM-MSC-derived conditioned medium
MSC-CM: Mesenchymal stem cell-conditioned medium
AF-MSCs: Human amniotic fluid-derived mesenchymal stem cells
JmjC: Jumonji C
TET: Ten-eleven translocation
5mC: 5-methylcytosine
5hmC: 5-hydroxymethylcytosine
CoCl_2_: Cobalt chloride
